# Detection of African Swine Fever Virus Antibodies in Serum and Oral Fluid Specimens Using a Recombinant Protein 30 (p30) Dual Matrix Indirect ELISA

**DOI:** 10.1371/journal.pone.0161230

**Published:** 2016-09-09

**Authors:** Luis G. Giménez-Lirola, Lina Mur, Belen Rivera, Mark Mogler, Yaxuan Sun, Sergio Lizano, Christa Goodell, D. L. Hank Harris, Raymond R. R. Rowland, Carmina Gallardo, José Manuel Sánchez-Vizcaíno, Jeff Zimmerman

**Affiliations:** 1 College of Veterinary Medicine, Iowa State University, Ames, Iowa, United States of America; 2 VISAVET Center and Animal Health Department, University Complutense of Madrid, Spain; 3 Harrisvaccines, Inc, Ames, Iowa, United States of America; 4 College of Liberal Arts and Sciences, Iowa State University, Ames, Iowa, United States of America; 5 IDEXX Laboratories, Westbrook, Maine, United States of America; 6 College of Veterinary Medicine, Kansas State University, Manhattan, Kansas, United States of America; 7 CISA-INIA, Madrid, Spain; Instituto Butantan, BRAZIL

## Abstract

In the absence of effective vaccine(s), control of African swine fever caused by African swine fever virus (ASFV) must be based on early, efficient, cost-effective detection and strict control and elimination strategies. For this purpose, we developed an indirect ELISA capable of detecting ASFV antibodies in either serum or oral fluid specimens. The recombinant protein used in the ELISA was selected by comparing the early serum antibody response of ASFV-infected pigs (NHV-p68 isolate) to three major recombinant polypeptides (p30, p54, p72) using a multiplex fluorescent microbead-based immunoassay (FMIA). Non-hazardous (non-infectious) antibody-positive serum for use as plate positive controls and for the calculation of sample-to-positive (S:P) ratios was produced by inoculating pigs with a replicon particle (RP) vaccine expressing the ASFV p30 gene. The optimized ELISA detected anti-p30 antibodies in serum and/or oral fluid samples from pigs inoculated with ASFV under experimental conditions beginning 8 to 12 days post inoculation. Tests on serum (n = 200) and oral fluid (n = 200) field samples from an ASFV-free population demonstrated that the assay was highly diagnostically specific. The convenience and diagnostic utility of oral fluid sampling combined with the flexibility to test either serum or oral fluid on the same platform suggests that this assay will be highly useful under the conditions for which OIE recommends ASFV antibody surveillance, i.e., in ASFV-endemic areas and for the detection of infections with ASFV isolates of low virulence.

## Introduction

African swine fever (ASF) is a highly contagious disease of pigs caused by a large, icosahedral, enveloped, double-stranded DNA virus belonging to family *Asfarvidiridae* [1]. ASF virus (ASFV) is listed by the World Organisation for Animal Health (OIE) as a notifiable disease and is classified as a select agent by authorities in the United States [2]. Infection with ASF virus (ASFV) can produce clinical signs ranging from sudden death to subacute and chronic disease, depending on the virulence of the isolate, the affected host, dose and route of infection [3]. ASFV is of major concern because of its high mortality rate, its severe economic impact on affected countries, and its recent rapid geographic expansion [4]. In particular, the expansion of ASFV within Africa and its introduction and spread across Transcaucasia, the Russian Federation, and Eastern Europe has heightened concerns for the emergence of the virus in ASFV-free countries either through infected free-ranging European wild boar (*Sus scrofa)*, illegal imports of pork and other derived products or through contaminated fomites moving in global trade networks [5,6,7].

Prevention of ASF is based on enhancing biosecurity on border controls, avoiding imports from infected countries, and creating barriers to avoid the introduction of the virus. Having entered a region, control and elimination relies on a timely and efficient process of ASFV detection, laboratory confirmation, and removal of infected animals [3]. In particular, antibody-based surveillance is useful in ASFV-endemic areas and for incursions involving low virulence ASFV isolates [2]. In the absence of vaccine, antibodies are a definitive indication of ASFV infection, are detectable for a prolonged period of time [4], and antibody assays are cost-effective and highly reproducible among laboratories. Surveillance programs in the Iberian Peninsula and Sardinia have used ASFV antibody detection in tandem with viral antigen detection, particularly for the detection of ASFV carrier animals and elucidating the epidemiological characteristics of the epidemics, i.e., time since the virus introduction into a premises [4].

A major impediment to the implementation of active surveillance is the cost of collecting and testing sufficient numbers of samples. Previous studies demonstrated the utility of oral fluids for the detection of a variety of swine pathogens including PRRSV [8,9], porcine circovirus type 2 [10], influenza A virus [11,12], and others [13]. In contrast to serum, oral fluid collection requires little labor, is stress-free ("welfare-friendly") for both animals and humans, and is more sensitive than individual pig samples for detecting infections in populations [14]. Previous research showed that ASFV antibodies could be detected in oral fluids and suggested that oral fluids could serve as a suitable diagnostic specimen for ASFV surveillance [15]. The objective of the present study was to develop a prototype indirect ELISA capable of detecting ASFV antibody either in serum or oral fluid specimens using safe (non-infectious) reagents, i.e., positive controls and target antigens.

## Material and Methods

### Experimental design

A recombinant protein 30 (p30) indirect ELISA for the detection of ASFV antibodies in either serum or oral fluid specimens was developed. The antigen used in the ELISA was selected by evaluating the serum antibody response of ASFV-infected pigs against three recombinant antigens (p30, p54, p72) using a multiplex fluorescent microbead-based immunoassay (FMIA; Luminex® Corporation, Austin TX USA). Non-hazardous ASFV antibody-positive control serum was created by inoculating pigs with a replicon particle (RP) vaccine expressing the ASFV p30 gene (Harrisvaccines, Inc., Ames IA USA). The antibody kinetics of the optimized ELISA were evaluated using serum and oral fluid collected over time post inoculation from animals (n = 17) inoculated with ASFV NHV/P68 isolate under experimental conditions. The absence of cross-reactivity was evaluated by testing ASFV p54 and p72 antibody positive serum generated by vaccinating pigs with RP vaccines expressing ASFV p54 and p72 genes. Diagnostic specificity of the assay was evaluated using serum (n = 200) and oral fluid (n = 200) samples from animals in commercial swine farms known to be free of ASFV infection.

### Samples from ASFV-inoculated animals

ASFV NHV/P68 (NHV), a low-virulent, non-hemadsorbing, p72 genotype I isolate was used in the *in vivo* studies. The virus was obtained from the European Union Reference Laboratory for ASF [Centro de Investigación en Sanidad Animal, Instituto Nacional de Tecnología Agraria y Alimentaria (CISA-INIA)] and was propagated and titrated as described by Carrascosa et al. (2011) [16].

Pigs were inoculated with NHV/P68 in biosafety level 3 (BSL3) animal facilities at Centro de Investigación en Sanidad Animal, belonging to Instituto Nacional de Investigación y Tecnología Agraria y Alimentaria (INIA, Valdeolmos, Madrid, Spain) as previously described (Code id. # ES281620002741) [15]. The studies were approved by the Dirección General del Medio Ambiente, Consejeria de Medio Ambiente y Ordenación del territorio (Comunidad de Madrid, Spain) and monitored by the INIA Regulatory Body for ethic and animal care (ORCEEA). The studies were performed in accordance with EC Directive 86/609/EEC and followed the recommendations provided in 2007/526/EC regarding the accommodation and care of animals used for experimental and other scientific purposes. In case of pain or distress any action was postponed until normal vital constant be recovered (et seq., extraction, sampling or inoculation). When signs of distress due to infection were evident, animals were examined daily following endpoint criteria i.e., 1) posture of the animal: if observed for a long time (> 48 h) immobility, or the animal cannot stand up, ataxia; 2) Severe anorexia: acute weight loss; 3) behavior: severe seizures, or persistent loss of movements control (> 24 h); 4) appearance and presence of abnormal secretions presence of severe bleeding in stool. Unprogrammed euthanasia was applied when circumstances warranting the endpoint criteria were given.

In experiment 1, 9 2-month-old Landrace x Large White pigs were each IM inoculated with a 1 ml of solution containing a virus concentration of 1 x 10^5^ 50% tissue culture infective doses (TCID_50_) per ml. Serum and oral fluid samples were collected periodically from day post inoculation (DPI) 0 through 26, using standard techniques [17]. In experiment 2, 4 2-month-old Landrace x Large White pigs were intramuscularly (IM) administered 1 ml of an inoculum with a virus concentration of 1 x 10^3^ TCID_50_ per ml and 4 animals were inoculated with 1 ml of an inoculum containing 1 x 10^5^ TCID_50_ per ml. Seroconversion was confirmed in both experiment 1 and 2 between 8–11 DPI using the OIE-approved ELISA based on a mixture of ASFV-derived antigens [2], and the INgezim PPA ELISA based on p72 protein (Ingenasa, Madrid, Spain).

### Candidate ASFV antigens

Three candidate recombinant antigens (p30, p54, and p72), were evaluated for use in the ELISA. ASFV (isolate BA71V) genes p30, p54 and p72 were commercially produced (GenScript, Pistacaway, NJ, USA) and cloned into a pHUE plasmid. This *Escherichia coli* (*E*. *coli*) expression vector provided for the over-expression of the polypeptides as histidine (his)-tagged ubiquitin fusion proteins [18]. The constructs were analyzed by restriction enzyme digestion and DNA sequencing, and then transformed into *E*. *coli* BL21(DE3)pLysS (Rosetta cells) (Invitrogen™, Carlsbad, CA, USA) for expression. The bacterial clone was grown in Luria-Bertani medium (Invitrogen™) containing 100 μg per ml ampicillin at 37°C. The over-expression of p30, p54, and p72 was induced by adding 1 mM isopropyl-β-thio-D-galactopyranoside (IPTG) at the mid-exponential phase (A_600_ between 0.4–0.6) followed by 3 additional h of incubation. Recombinant his-tagged fusion proteins p30 and p54, and his-tagged p72 over-expressed in inclusion bodies, were purified from clarified extracts of Rosetta cells under denaturing conditions (8 M urea) using a nickel-nitrilotriacetic acid (Ni-NTA) chelate affinity chromatography kit (PrepEase® His-tagged protein purification kit, USB Corporation, Cleveland, OH, USA) performed according to manufacturer’s instructions. Briefly, frozen pellets obtained from cultures of Rosetta cells containing p30, p54, and p72 genes cloned into the pHUE expression vector were thawed and resuspended in 1 × LEW lysis buffer (USB Corporation) containing 8 M urea (Sigma-Aldrich, St. Louis, MO, USA). Cells were lysed by sonication for 10 cycles of 20s on ice using a Vibra-Cell™ sonicator (Sonics and Materials, New Town, CT, USA). The crude extracts were centrifuged at 50,000 × g for 30 min at 4°C and the supernatants were purified by Ni-NTA affinity chromatography. The purified antigens were analyzed and their apparent molecular mass was determined by 12% sodium dodecyl sulfate-polyacrylamide gel electrophoresis (SDS-PAGE).

### Multiplex fluorescent microbead-based immunoassay (FMIA)

ELISA antigen selection was done by evaluating the serum antibody response of ASFV-inoculated animals as described above (samples from ASFV-inoculated animals) against p30, p54, and p72 using a multiplex fluorescent microbead-based immunoassay (FMIA; Luminex Corp., Austin, TX, USA). The FMIA was performed as described elsewhere [19]. In brief, recombinant antigens p30, p54, and p72 were purified under denaturing conditions and then precipitated to remove the urea and exchange the buffer. One volume of 8M urea buffer containing either recombinant p30, p54, or p72 polypeptides was added to nine volumes of ice-cold 100% ethanol, mixed gently, incubated for 2 h at -20°C, and centrifuged (90 s at 7,000 × *g*). Thereafter, the pellet was washed with nine volumes of 90% ice-cold ethanol and resuspended in 1X phosphate buffered saline (PBS) with 0.1% SDS. The covalent coupling of microbead sets, individually identifiable by unique fluorescence, to purified recombinant p30, p72, and p54 polypeptides was performed as previously reported using a two-step carbodiimide reaction [19,20]. A total of 50 μg (50 μg antigen per 5 x 10^6^ beads) of each antigen [p30 (bead region 12), p54 (bead region 20), and p72 (bead region 64)] were coupled to 2.5 x 10^6^ carboxilated fluorescent microbeads. To perform the 3-plex FMIA, antigen-coupled beads were sonicated, mixed by vortexing, and diluted in a storage buffer to a final concentration of ~2,500 beads per well for each recombinant protein. Serum samples were diluted 1:25 in assay buffer (0.1 M PBS, 10% goat serum [Gibco®, Life Technologies, Grand Island, NY, USA], 0.05% Tween-20, pH 7.2) and mixed with 50 μl of the bead suspension in each well (Bio-Plex Pro™ flat bottom plates, Bio-Rad Laboratories Inc., Hercules, CA, USA). The plates were incubated for 30 min on a micro plate shaker (VWR®, Radnor, PA, USA) set at 500 rpm and washed three times with 200 μl of 0.1 M PBS containing 0.05% Tween-20 (PBST). This and other incubations were performed at ~22°C in a dark environment. Next, 50 μl of biotin-labelled Protein A (Sigma-Aldrich, St Louis, MO, USA) in assay buffer (1:100 dilution) was added to each well, followed by a 30 min incubation. After plates were washed 3 times with PBST, 50 μl of streptavidin phycoerythrin (SAPE; 2.5 μg/ml in assay buffer) was added to each well and the plates incubated for 30 min. After an additional wash step, the beads were resuspended in 100 μl of assay buffer and analyzed using a dual-laser Bio-Plex® 200 instrument (Bio-Rad Laboratories, Inc.). Events were gated to exclude doubles and other aggregates (Bio-Plex Manager™ software 6.0, Bio-Rad Laboratories, Inc.).

The data were estimated from at least 50 beads and reported as median fluorescent intensities (MFI). Two "background wells" (coupled microspheres incubated with serum diluent in the absence of sample) were included on each plate to measure non-specific binding of the detector. The MFI response was corrected by subtracting the background well signal (the mean of the two wells) from the signal obtained for each individual sample.

### Recombinant p30 indirect ELISA

ELISA conditions, e.g., p30 coating and blocking conditions, sample and conjugate dilutions, buffers, and incubation times were concurrently optimized for antibody detection in serum and oral fluid specimens. The reagents used in the assay, i.e., sample and conjugate diluents, substrate, stop solution, and wash solution, were provided by a commercial entity (IDEXX Laboratories, Inc., Westbrook, ME USA).

To prepare plates, 96-well microtitration plates (Nunc, Thermo Fisher Scientific, Agawam, MA, USA) were manually coated with 100 μl of p30 polypeptide per well at a concentration of 5 μg per ml in phosphate-buffered saline (PBS) pH 7.4 (Gibco®, Life Technologies) and were then incubated at 4°C overnight. Following incubation, plates were washed 5 times, blocked with a solution (300 μl per well) containing 1% bovine serum albumin (Jackson ImmunoResearch Inc.), and incubated at 25°C for 2 h. Plates were then dried at 37°C for 4 h and stored at 4°C in a sealed bag with desiccant packs. Plate lots with a coefficient of variation ≥10% were rejected.

#### ELISA negative and positive plate controls

A bank of serum and oral fluid samples containing antibodies specific for ASFV p30, p54, and p72 was established by vaccinating pigs with replicon particle (RP) vaccines expressing genes for ASFV p30, p54, and/or p72 antigens (Harrisvaccines Inc., Ames, IA, USA).

Replicon particles are single-cycle RNA vectors capable of expressing foreign antigens *in vivo* [21]. To prepare RP vaccines, ASFV (strain BA71V) genes p30, p54, and p72 were *de novo* synthesized, codon-optimized for expression in swine (GenScript), and then modified by PCR to add appropriate restriction sites and a C-terminal 6x-histidine tag sequence. The modified genes were cloned into a replicon vector plasmid derived from Venezuelan equine encephalitis virus (VEEV) strain TC-83 [22]. Thereafter, replicon vector and TC-83 helper plasmids were transcribed *in vitro* using T7 RNA polymerase [22]. RPs were generated by electroporation of p30, p54, and/or p72 replicon RNA and structural gene helper RNAs into Vero cells. The RPs were harvested from culture fluids and then purified by size exclusion/ionic exchange filtration [23]. Vero cell monolayers were then infected with p30 RP, p54 RP, p72 RP or a combination of the three. The expression of p30, p54, and p72 was confirmed by western blot analysis on RP-infected cell lysates using anti-His monoclonal antibodies. The infectious titer of the purified bulk RPs was determined by immunofluorescence assay (IFA) and expressed as immunofluorescence units (IU) per ml [22].

Thereafter, 39 12-week-old pigs were randomly allocated to one of 5 RP vaccine groups: (1) p30 RP vaccine, (2) p54 RP vaccine, (3) p72 RP vaccine, (4) p30/54/72 RP vaccine, and (5) unvaccinated controls. The vaccines were intramuscularly inoculated at days post vaccination (DPV) 0 and 21 with 2 ml of a solution containing 5 x 10^8^ IU per ml of the designated RP except the p30/54/72 group received 2 ml of a solution containing 1.5 x 10^9^ IU per ml. Antibody responses against p30, p54, and p72 were monitored over time by FMIA using pen-based oral fluid samples (collected daily) and individual pig serum samples (collected DPV 0, 6, 14, 21, and 28). At DPV 28, all animals were humanely euthanized and exsanguinated. The harvested serum was pooled by RP vaccine group, processed, and stored at -80°C. ELISA negative and positive plate controls consisted of pooled sera from p30 RP-vaccinated and non-vaccinated groups, respectively.

#### ELISA procedure

Serum samples were diluted 1:100 and oral fluid samples were diluted 1:2, after which plates were loaded with 100 μl of diluted sample per well. Antibody-positive and negative controls were run in duplicate on each ELISA plate. Plates were incubated at 25°C for 1 h and then washed 5 times. Thereafter, 100 μl of peroxidase conjugated goat anti-swine IgG (H+L) antibody (Jackson ImmunoResearch, Inc.) diluted 1:60,000 for serum or 1:25,000 for oral fluid was added to each well and the plates incubated at 25°C for 30 min. After a washing step, the peroxidase reaction was visualized by adding 100 μl of tetramethylbenzidine-hydrogen peroxide (TMB) substrate solution per well. Plates were incubated at room temperature for 10 min, 50 μl of stop solution was added, and the plates were read immediately thereafter. Reactions were measured as optical density (OD) at 450 nm using an ELISA plate reader (Biotek® Instruments Inc., Winooski, VT) operated with commercial software (GEN5TM, Biotek® Instruments Inc.). Serum and oral fluid antibody responses were expressed as sample-to-positive (S/P) ratios:

S/Pratio=(sampleOD−negativecontrolmeanOD)/(positivecontrolmeanOD−negativecontrolmeanOD)

#### Samples from ASFV-negative animals

The Western Hemisphere is currently recognized as free of ASFV. Therefore, the specificity of the ELISA was evaluated using samples submitted to the Iowa State University Veterinary Diagnostic Laboratory (Ames, Iowa USA) for routine diagnostic testing from commercial swine herds in North America. Samples included 200 serum samples from pigs 3 to 10 weeks of age and 200 oral fluid samples from pigs 3 weeks to 7 months of age.

### Analysis

Statistical analyses were performed using commercial software (SAS® 9.3, SAS® Institute Inc., Cary, NC).

The antigen for the ASFV indirect ELISA was selected by evaluating the serum antibody response against p30, p54, and p 73 in 9 pigs inoculated with NHV/P68 (experiment 1) and selecting the antigen that provided the greatest differentiation. After testing by multiplex FMIA, the MFI serum antibody responses were analyzed using a generalized linear mixed model with DPI as a repeated measure for each pig. Post-hoc pairwise comparisons among p30, p54, and p72-specific responses were done at each DPI. In addition, the antigen-specific antibody results for each DPI were compared to day 0.

The serum antibody response in pigs inoculated with replicon particle (RP) vaccines expressing p30, p54, and/or p72 genes, was measured by FMIA and p30 indirect ELISA. The data were analyzed using generalized linear mixed models with DPV as a repeated measure for each pig and post hoc pairwise comparisons performed for each DPV. FMIA assay serum antibody FMI results were compared among 5 groups: (1) p30 RP vaccine, (2) p54 RP vaccine, (3) p72 RP vaccine, (4) p30/54/72 RP vaccine, and (5) unvaccinated controls. For the p30 indirect ELISA, serum and oral fluid antibody S/P results were compared among 3 groups: (1) p30 RP vaccine, (2) p30/54/72 RP vaccine, and (3) a control group defined as unvaccinated pigs (one pen of 4 pigs) and pigs inoculated with RP vaccines expressing ASFV p54 (one pen of 8 pigs) or p72 (one pen of 8 pigs). Because one pen-based oral fluid sample was collected from each treatment each day, the data were re-defined to permit statistical analysis. That is, data from DPV 0 to 7 were classified as Week 1, data from DPV 8 to 21 as Week 2/3, and DPV 22 to 28 as Week 4. The oral fluid p30 indirect ELISA data Week 1, 2/3, and 4 were then compared using a one-way ANOVA. Thereafter, the serum and oral fluid p30 indirect ELISA S/P responses in 17 pigs (experiments 1 and 2) inoculated with ASFV NHV/P68 were analyzed using a generalized linear mixed model with DPV as a repeated measure for each pig and experiment. Post hoc pairwise comparisons performed for each DPV.

## Results

### Serum antibody responses to ASFV inoculation by multiplex FMIA (Fig 1)

**Fig 1:**
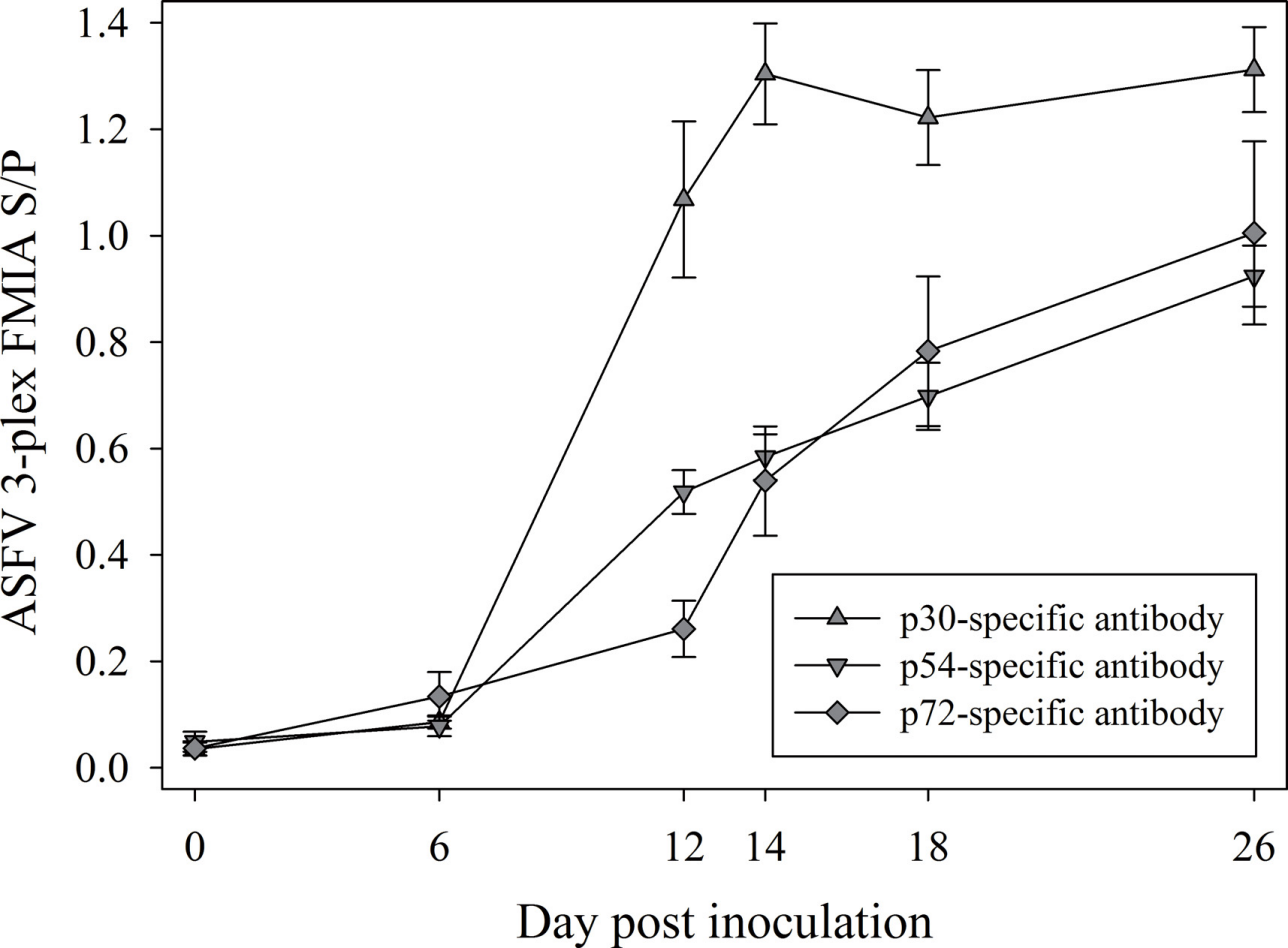
ASFV multiplex fluorescent microbead-based immunoassay (FMIA) sample-to-positive (S/P) serum antibody response (mean, SE) against three recombinant antigens (p30, p54, p72) in 9 pigs inoculated with ASFV NHV/P68 (experiment 1).

The reactivity of three major recombinant ASFV antigens (p30, p72 and p54) was evaluated by testing serum samples collected between 0 and 26 DPI from 9 pigs inoculated with ASFV isolate NHV/P68 (experiment 1) in the multiplex FMIA. As shown in Fig 1, antibody responses were observed over time for all 3 individual ASFV antigens (p30, p54, and p72). Comparisons showed that p30 and p54 responses at DPI ≥ 12 were higher than DPI 0 (*p* < 0.0001) and DPI 6 (*p* < 0.0001) responses. The response against p72 was slower than p30 and p54 responses, however, p72 FMIA responses at DPI 12 were significantly higher than DPI 0 (*p* = 0.0322), as were responses at DPI ≥ 14 (*p* < 0.0001). In particular, the markedly higher serum IgG median fluorescent intensity against p30 relative to p54 and p72 indicated that this antigen was the best candidate for the development of a dual matrix serum/oral fluid indirect ELISA.

### Serum antibody responses to RP vaccines by multiplex FMIA (Table 1)

Analysis of the multiplex FMIA protein-specific serum antibody responses in RP vaccine groups (1) p30 RP vaccine, (2) p54 RP vaccine, (3) p72 RP vaccine, (4) p30/54/72 RP vaccine, and (5) unvaccinated controls found no difference in MFI responses at DPV 0 and 6 (*p* > 0.05). At DPVs 14, 21, and 28, the p30/54/72 RP vaccine MFI responses were significantly higher than all other treatments (*p* < 0.001), except the p72 RP MFI response against p72 at DPV 14 (*p* = 0.098). All homologous combinations of RP vaccine and FMIA ASFV antigen produced higher MFI responses than heterologous combinations (*p* < 0.001). No differences in MFI response were detected among heterologous combinations of RP vaccine and FMIA antigen or negative controls (*p* > 0.05).

**Table 1:** African swine fever virus (ASFV) antigen-specific serum antibody responses detected by multiplex fluorescent microbead-based immunoassay (FMIA) in pigs inoculated with replicon particle (RP) vaccines expressing p30, p54, and/or p72 genes.

RP vaccine groups^a^	FMIA ASFV recombinant antigen	Mean (SE) median fluorescent intensity (MFI) by day post vaccination (DPV)				
		DPV 0^b^	DPV 6^b^	DPV 14^c^	DPV 21^c^	DPV 28^c^
1. p30 (8 pigs)	**p30 (homologous)**	**0.062 (0.010)**	**0.083 (0.010)**	**0.387 (0.010)**	**0.432 (0.010)**	**1.410 (0.010)**
	p54	0.062 (0.006)	0.065 (0.006)	0.065 (0.006)	0.065 (0.006)	0.066 (0.006)
	p72	0.063 (0.007)	0.065 (0.007)	0.064 (0.007)	0.065 (0.007)	0.065 (0.007)
2. p54 (8 pigs)	p30	0.059 (0.010)	0.059 (0.010)	0.061 (0.010)	0.060 (0.010)	0.062 (0.010)
	**p54 (homologous)**	**0.062 (0.006)**	**0.063 (0.006)**	**0.268 (0.006)**	**0.350 (0.005)**	**0.967 (0.006)**
	rp72	0.064 (0.007)	0.065 (0.007)	0.068 (0.007)	0.067 (0.007)	0.068 (0.007)
3. p72 (8 pigs)	p30	0.061 (0.010)	0.061 (0.010)	0.068 (0.010)	0.067 (0.010)	0.066 (0.010)
	p54	0.063 (0.006)	0.063 (0.006)	0.066 (0.006)	0.066 (0.006)	0.066 (0.006)
	**p72 (homologous)**	**0.063 (0.007)**	**0.070 (0.007)**	**0.322 (0.007)**	**0.392 (0.007)**	**1.140 (0.007)**
4. p30/54/72 (4 pigs)	**p30 (homologous)**	**0.062 (0.013)**	**0.070 (0.013)**	**0.548 (0.013)**	**0.582 (0.013)**	**1.670 (0.013)**
	**p54 (homologous)**	**0.064 (0.008)**	**0.068 (0.008)**	**0.351 (0.008)**	**0.531 (0.008)**	**1.179 (0.008)**
	**p72 (homologous)**	**0.068 (0.010)**	**0.085 (0.010)**	**0.343 (0.010)**	**0.513 (0.010)**	**1.251 (0.010)**
5. Unvaccinated controls (4 pigs)	p30	0.069 (0.013)	0.069 (0.013)	0.062 (0.015)	0.063 (0.015)	0.062 (0.015)
	p54	0.068 (0.008)	0.068 (0.008)	0.064 (0.010)	0.064 (0.001)	0.065 (0.001)
	p72	0.068 (0.010)	0.067 (0.010)	0.064 (0.012)	0.067 (0.012)	0.068 (0.012)

^a^ Pigs were intramuscularly inoculated at 0 and 21 DPV with 2 ml of a solution containing 5 x 10^8^ IU per ml of the designated RP except the p30/54/72 group received 2 ml of a solution containing 1.5 x 10^9^ IU per ml.

^b^ DPV 0, 6: No difference in response was detected among groups (*p* > 0.05).

^c^ DPV 14, 21, 28: Group 4 responses were significantly higher than all other treatments (*p* < 0.001), except the Group 3 homologous response at DPV 14 (*p* = 0.098). All homologous (RP vaccine-FMIA antigen) combinations produced higher responses than heterologous combinations (*p* < 0.001). No differences in response were detected among heterologous RP vaccine-FMIA antigen combinations and negative controls (*p* > 0.05).

### Serum (Fig 2A) and oral fluid (Fig 2B) antibody responses to RP vaccines by p30 indirect ELISA

No difference was found in the serum S/P responses on DPI 0 or 6 (*p* > 0.05) among (1) p30 RP vaccine, (2) p30/54/72 RP vaccine, and (3) control groups. At DPV 14, 21, and 28, the p30 RP and p30/54/72 RP vaccine groups had significantly higher S/P responses than controls (*p* < 0.0001). Further, the p30/54/72 RP vaccine produced significantly higher S/P responses than the p30 RP vaccine on these same time points (*p* < 0.04).

**Fig 2:**
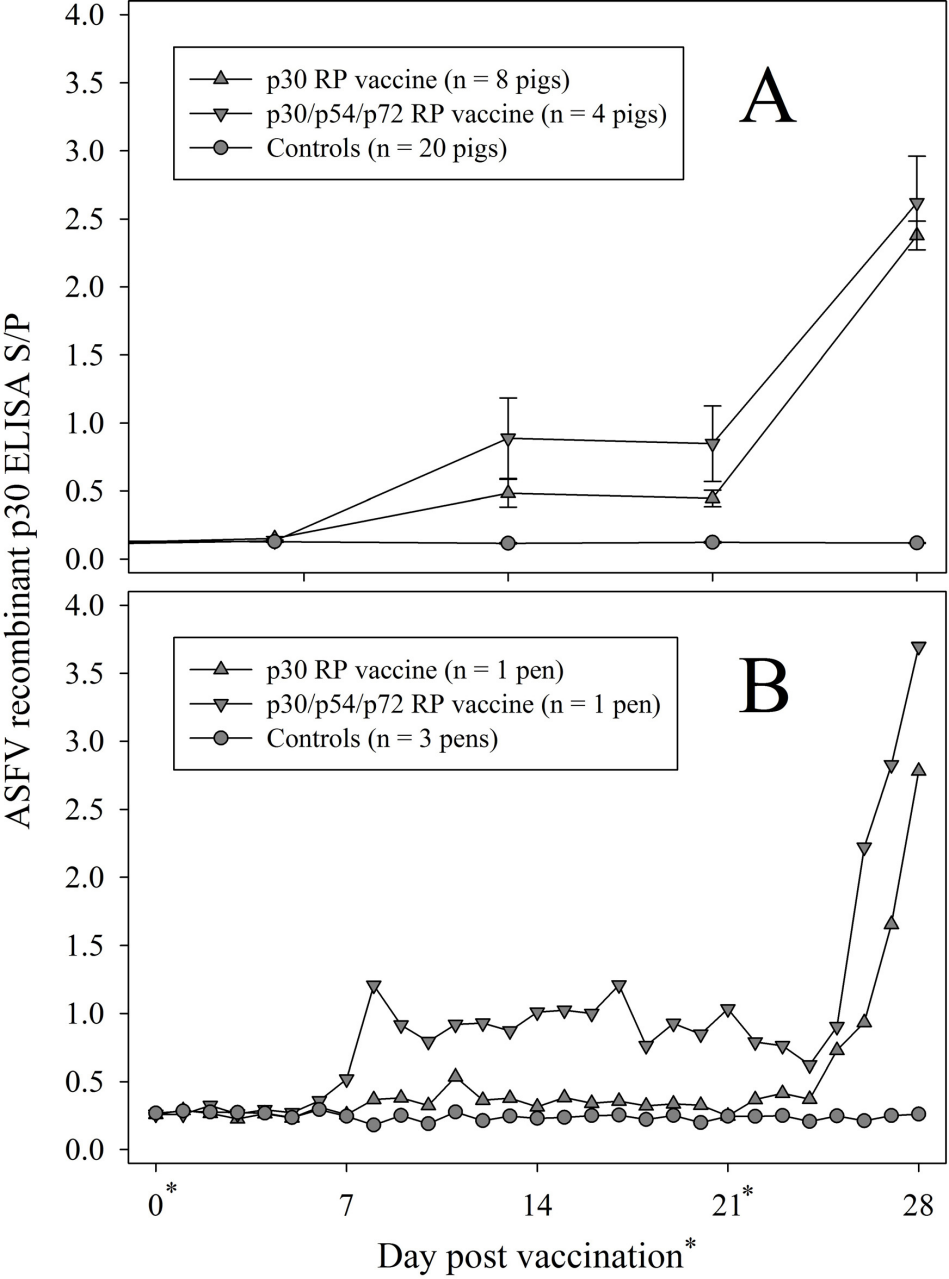
**ASFV recombinant p30 antibody ELISA sample-to-positive (S/P) responses in (2A) individual pig serum (mean, SE) and (2B) pen-based oral fluid samples following inoculation with replicon particle (RP) vaccines expressing ASFV p30 or a combination of ASFV p30/54/72.** Controls were defined as unvaccinated pigs (one pen of 4 pigs) and pigs inoculated with RP vaccines expressing ASFV p54 (one pen of 8 pigs) or ASFV p72 (one pen of 8 pigs). In all cases, pigs received two doses of vaccine (days 0 and 21).

The recombinant p30 indirect ELISA response in oral fluids is given in Fig 2B. Recollect that, although the samples were collected daily, the data were reclassified by week to perform the analysis. No difference was found in the oral fluid S/P responses in Week 1 (*p* > 0.05) among (1) p30 RP vaccine, (2) p30/54/72 RP vaccine, and (3) control groups. At Week 2/3, the p30/54/72 RP vaccine groups had signficantly higher S/P responses than either the p30 group or the control group (*p* < 0.0003), with no difference detected between the p30 and control groups (*p* > 0.05). At Week 4, the p30/54/72 RP vaccine response was higher than both the p30 RP and control groups (p < 0.006) and the p30 response was higher than the control group (p = 0.0008).

### Serum and oral fluid antibody responses to ASFV inoculation by p30 indirect ELISA (Fig 3)

Analyses of ASFV p30 antibody ELISA responses were performed on testing results from serum and oral fluid samples collected from 17 pigs (experiment 1 and 2) inoculated with ASFV isolate NHV/P68. The analysis showed that serum antibody S/P responses at DPI ≥ 6 were significantly greater than DPI 0 (*p* < 0.03), whereas oral fluid antibody S/P responses were significantly greater than DPI 0 at DPI 8 and later (*p* < 0.001). Comparisons of serum vs. oral fluid ELISA S/P data showed no significant differences in the response except on DPIs 12 and 18 (*p* ≤ 0.01).

**Fig 3:**
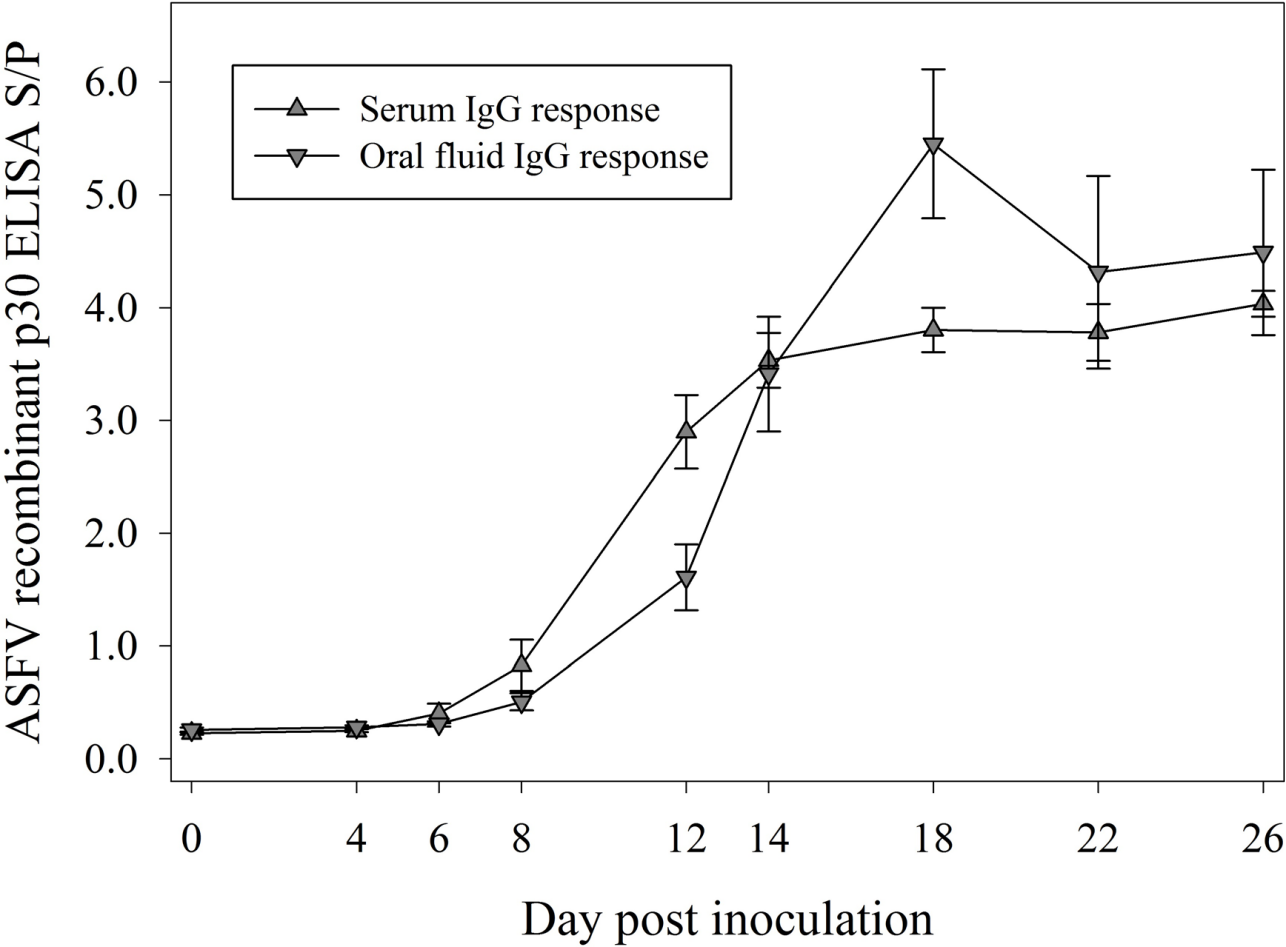
ASFV recombinant p30 antibody ELISA serum-to-positive (S/P) responses in serum (mean, SE) and oral fluid (mean, SE) samples from 17 pigs (experiments 1 and 2) inoculated with ASFV NHV/P68.

### Performance of the p30 dual-matrix indirect ELISA (Fig 4)

**Fig 4:**
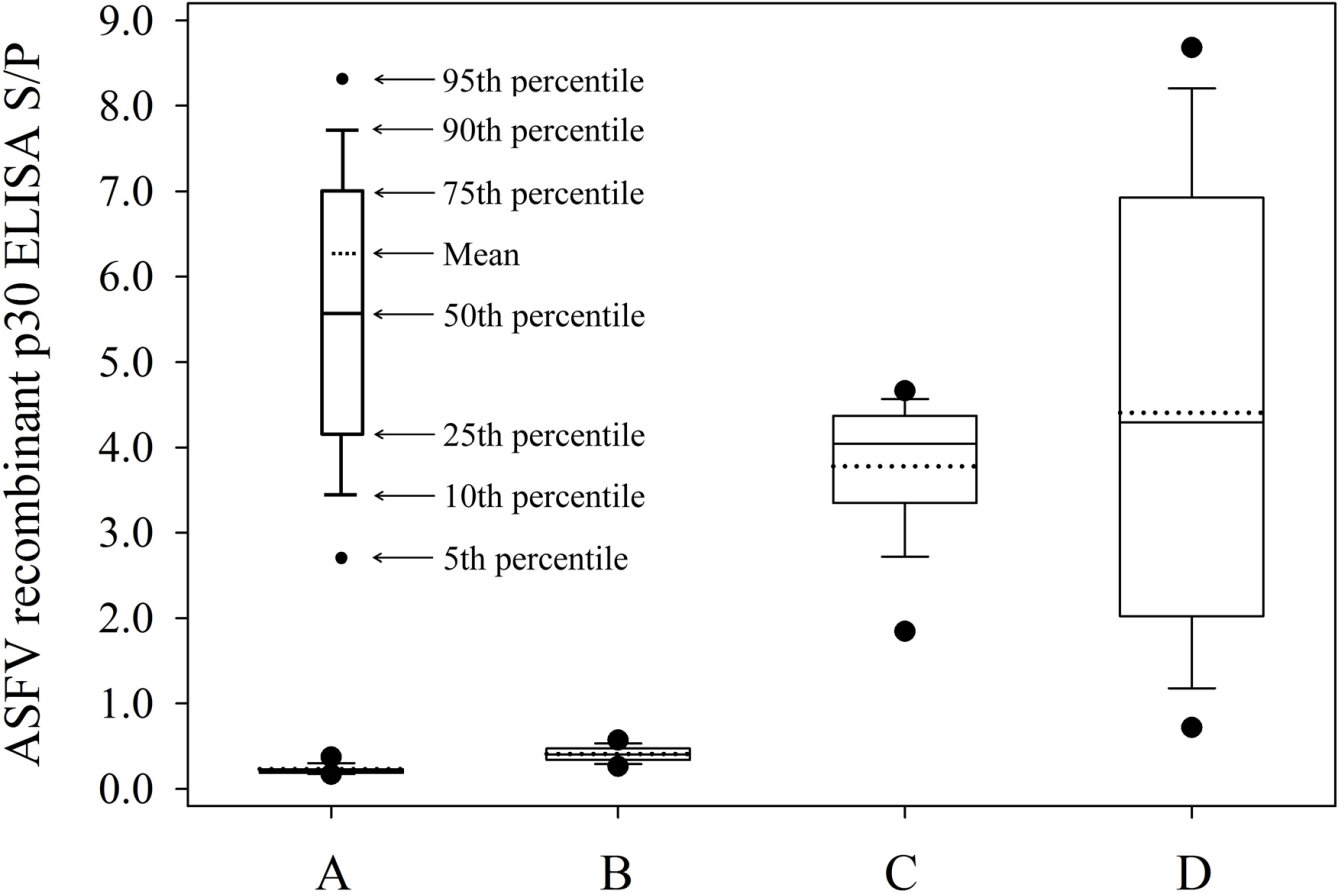
**Distribution of ASFV recombinant p30 antibody ELISA serum-to-positive (S/P) responses in (A) serum (n = 200) and (B) oral fluid (n = 200) specimens from North American commercial pigs and (C) serum (n = 52) and (D) oral fluid (n = 46) collected ≥ 14 days following inoculation with ASFV NHV/P68**.

The diagnostic performance of the dual-matrix serum/oral fluid p30 antibody ELISA was assessed by analyzing the distribution of ASFV recombinant p30 antibody ELISA S/P responses in serum (n = 200) and oral fluid (n = 200) specimens of known negative ASFV status from North American commercial pigs and serum (n = 52) and oral fluid (n = 46) collected ≥ 14 days following inoculation with ASFV NHV/P68. The box-and-whisker plots shown in Fig 4 visually describe the distribution of the data. Analysis of the data provided the estimates of S/P means (95% confidence intervals): negative serum (mean S/P 0.23, 95% CI: 0.21, 0.25), negative oral fluid (mean S/P 0.41, 95% CI: 0.39, 0.42), positive serum (mean S/P 3.80, 95% CI: 3.56, 4.00), and positive oral fluids (mean S/P 4.41, 95% CI: 3.65, 5.16).

## Discussion

The current epidemiological situation of ASF in the Caucasus and the Russian Federation including the incursion of the disease in Belarus, Ukraine, and Eastern Europe, indicates that further geographic spread of ASF is likely to occur [3]. Given this scenario, continuous improvements in ASF diagnosis and surveillance are critical for disease containment. In endemic situations, recovered ASFV carrier pigs and persistently infected wild pigs constitute the biggest challenge in controlling the disease. Pigs that survive natural infection develop antibodies against ASFV from 7 to 10 days after infection that are detectable for a prolonged period of time [7,24]. Therefore, ASFV surveillance is vital but a simpler, more cost-effective approach must be developed based on efficient, low-cost specimen collection, accurate diagnostic testing, and no-infectious material.

Current ASFV antibody-based tests approved by the World Organization for Animal Health (OIE) are based on the use of serum as a test specimen and live virus as antigen, which necessitates level 3 biosafety facilities for propagation and handling of the live virus [2]. Antibody-based immunoassays based on recombinant antigens obviate the risk of handling live virus and facilitate high-throughput testing [25,26,27,28]. Furthermore, it has become widely recognized that oral fluid specimens are a viable option to serum, are easily collected, and provide diagnostic information equivalent or superior to serum samples [8,14]. An earlier study showed that ASFV antibodies could be detected in oral fluids and suggested that oral fluids could serve as a suitable specimen for ASFV surveillance [15]. Therefore, the aim of this study was to develop an ASFV dual matrix serum/oral fluid antibody ELISA based on non-infectious material and amenable to high-throughput testing.

Previous reports indicated ASFV proteins p30, p54, and p72 as likely diagnostic antigens [28,29]. To select the best candidate antigen, spectrally-distinguishable fluorescent beads-based xMAP^TM^ technology (Luminex®) were used as a screening tool to evaluate the reactivity of the ASFV recombinant p30, p54 and p72 proteins simultaneously in a single reaction, as described elsewhere [30]. Results on experimental samples showed that p30 provided the best diagnostic performance. These results corroborate previous reports on the utility of p30-based immunoassays for early detection of ASFV antibodies [27]. Therefore, p30 was selected for use in the ASFV dual-matrix serum/oral fluid indirect ELISA.

Antibodies against attenuated ASFV isolate NHV/P68 were detected by p30 indirect ELISA in either serum or oral fluid specimens run on the same plate with approximately equivalent performance, yet the assay was highly specific for both specimen types. These results represent an improvement in assay performance for oral fluid specimens compared to our previous report [15]. Notably, oral fluid antibodies were detected by this assay as early as 8 DPI, which is equivalent to the performance reported for the OIE indirect serum antibody ELISA [15]. These data showed that the p30 indirect ELISA could be useful in detecting ASFV-infected animals, even as early as 8 DPI. In the current study, it was not possible to estimate the optimal S/P cutoff and associated diagnostic sensitivity and specificity for the ELISA because of the availability of a limited number of known ASFV-positive specimens. However, testing of 200 serum samples and 200 oral fluid samples from known negative animals suggested that the test is highly diagnostically specific. Test specificity is of critical importance because false-positive results rapidly erode confidence in surveillance and eradication programs [27].

ASFV-antibody negative and antibody-positive plate controls are mandatory as part of the ELISA test development, but sera from ASFV-infected pigs are not an option in a commercial ELISA because it is a potential biohazard [4,31,32]. In this study, RP vaccines expressing ASFV p30, p54, or p72 genes were used to generate a bank of ASFV-antibody positive and negative oral fluid and serum samples for use as internal controls, thereby avoiding the use of infectious ASFV [22]. Data presented here demonstrate that RP expressing genes inserted in the alphavirus vector, i.e., p30, p54, p72, or a combination of the three, could induce dose-dependent immune responses specific to ASFV.

Overall, the results showed that the p30 indirect ELISA detects ASFV antibodies at early stages post-exposure in either oral fluid or serum samples. Given the increased surveillance efficiency provided by oral fluid sampling [14] and the ability to corroborate results using serum samples, the ASFV p30 antibody would be a highly useful under conditions that warrant ASFV surveillance.

## References

[1] Dixon LK, Escribano JM, Martins C, Rock DL, Salas ML, Wilkinson PJ. Asfarviridae In: Fauquet CM, Mayo MA, Maniloff J, Desselberger U, Ball LA, editors. Virus Taxonomy. VIII Report of the ICTV. London: Elsevier, Academic Press; 2005 pp. 135–143.

[2] Oura CAL, Arias M. African swine fever; 2012. Manual of Diagnostic Tests and Vaccines for Terrestrial Animals. OIE, World Organisation for Animal Health. Available: http://www.oie.int/international-standard-setting/terrestrial-manual/access-online/. Accessed 26 May 2015.

[3] Sánchez-Vizcaíno JM, Mur L, Gomez-Villamandos JC, Carrasco L. An update on the epidemiology and pathology of African swine fever. J Comp Path. 2015; 152: 9–21. 10.1016/j.jcpa.2014.09.003 25443146

[4] Arias M, Sánchez-Vizcaíno JM. African swine fever In: Zimmerman JJ, Karriker LA, Ramirez A, Schwartz KJ, Stevenson GW, editors. 10^th^ ed. Diseases of Swine. Hoboken, New Jersey: Wiley-Blackwell; 2012pp. 396–404.

[5] European Food Safety Authority (EFSA). Evaluation of possible mitigation measures to prevent introduction and spread of African swine fever virus through wild boar. EFSA Journal. 2014; 12(3): 3616.

[6] Mur L, Igolkin A, Varentsova A, Pershin A, Remyga S, Shevchenko I, et al Detection of African Swine Fever Antibodies in Experimental and Field Samples from the Russian Federation: Implications for Control. Transbound Emerg Dis. 2014; 11 30 10.1111/tbed.12304 25440300

[7] Sánchez-Vizcaíno JM, Mur L, Martínez-López B. African swine fever: An epidemiological update. Transbound Emerg Dis. 2012; 59: 27–35. 10.1111/j.1865-1682.2011.01293.x 22225967

[8] Kittawornrat A, Panyasing Y, Goodell C, Wang C, Gauger P, Harmon K, et al Porcine reproductive and respiratory syndrome virus (PRRSV) surveillance using pre-weaning oral fluid samples detects circulation of wild-type PRRSV. Vet Microbiol. 2014; 168: 331–339. 10.1016/j.vetmic.2013.11.035 24393634

[9] Kittawornrat A, Prickett J, Wang C, Panyasing Y, Ballagi A, Rice A, et al Detection of porcine reproductive and respiratory syndrome virus (PRRSV) antibodies in oral fluid specimens using a commercial PRRSV serum antibody ELISA. J Vet Diagn Invest. 2012; 24: 262–269. 10.1177/1040638711435679 22379043

[10] Prickett J, Simer R, Yoon KJ, Kim WI, Zimmerman J. Oral-fluid samples for surveillance of commercial growing pigs for porcine reproductive and respiratory syndrome virus and porcine circovirus type 2 infections. J Swine Health Prod. 2008; 16: 86–91.

[11] Prickett JR, Johnson J, Murtaugh MP, Puvanendiran S, Wang C, Zimmerman JJ, et al Prolonged detection of PCV2 and anti-PCV2 antibody in oral fluids following experimental inoculation. Transbound Emerg Dis. 2011; 58: 121–127. 10.1111/j.1865-1682.2010.01189.x 21223532

[12] Detmer SE, Patnayak DP, Jiang Y, Gramer MR, Goyal SM. Detection of influenza A virus in porcine oral fluid samples. J Vet Diagn Invest. 2011; 23: 241–247. 21398442 10.1177/104063871102300207

[13] Panyasing Y, Goodell C, Kittawornrat A, Wang C, Levis I, Defresne I, et al Influenza A virus surveillance based on pre-weaning piglet oral fluid. Transbound Emerg Dis. 2014; 10.1111/tbed.12307 25488821

[14] Prickett JR, Zimmerman JJ. The development of oral fluid-based diagnostics and applications in veterinary medicine. Anim Health Res Rev. 2010; 11: 207–216. 10.1017/S1466252310000010 20202287

[15] Olsen C, Wang C, Christopher-Hennings J, Doolittle K, Harmon K, Abate S, et al Probability of detecting PRRSV infection using pen-based swine oral fluid specimens as a function of within-pen prevalence. J Vet Diagn Invest. 2013; 25: 328–335. 10.1177/1040638713481471 23536612

[16] Mur L, Gallardo C, Soler A, Zimmerman J, Pelayo V, Nieto R, et al Potential use of oral fluid samples for serological diagnosis of African swine fever. Vet Microbiol. 2013; 165: 135–139. 10.1016/j.vetmic.2012.12.034 23374655

[17] Carrascosa AL, Bustos MJ, de Leon P. Methods for growing and titrating African swine fever virus: field and laboratory samples. Curr Protoc Cell Biol. 2011; Chapter 26: Unit 26.14. 10.1002/0471143030.cb2614s53 22161547

[18] Kittawornrat A, Engle M, Johnson J, Prickett J, Schwartz T, Whitney D, et al Porcine reproductive and respiratory syndrome virus (PRRSV) in serum and oral fluid samples from individual boars: Will oral fluid replace serum for PRRSV surveillance? Virus Res. 2010; 154: 170–176. 10.1016/j.virusres.2010.07.025 20670665

[19] Catanzariti AM, Soboleva TA, Jans DA, Board PG, Baker RT. An efficient system for high-level expression and easy purification of authentic recombinant proteins. Protein Sci. 2004; 13: 1331–1339. 15096636 10.1110/ps.04618904 PMC2286746

[20] Giménez-Lirola LG, Jiang YH, Sun D, Hoang H, Yoon KJ, Halbur PG, et al Simultaneous detection of antibodies against Apx toxins ApxI, ApxII, ApxIII, and ApxIV in pigs with known and unknown *Actinobacillus pleuropneumoniae* exposure using a multiplexing liquid array platform. Clin Vaccine Immunol. 2014; 21: 85–95. 10.1128/CVI.00451-13 24226091 PMC3910918

[21] Staros JV, Wright RW, Swingle DM. Enhancement by N-hydroxysulfosuccinimide of water-soluble carbodiimide-mediated coupling reactions. Anal Biochem. 1986; 156:220–222. 3740412 10.1016/0003-2697(86)90176-4

[22] Rayner JO, Dryga SA, Kamrud KI. Alphavirus vectors and vaccination. Rev Med Virol. 2002; 12: 179–296. 12211042 10.1002/rmv.360

[23] Kamrud KI, Custer M, Dudek JM, Owens G, Alterson KD, Lee JS, et al Alphavirus replicon approach to promoterless analysis of IRES elements. Virology. 2007; 360: 376–387. 17156813 10.1016/j.virol.2006.10.049 PMC1885372

[24] Hooper JW, Ferro AM, Golden JW, Silvera P, Dudek J, Alterson K, et al Molecular Smallpox Vaccine Delivered by Alphavirus Replicons Elicits Protective Immunity in Mice and Non-human Primates. Vaccine. 2009; 28: 494–511. 10.1016/j.vaccine.2009.09.133 19833247 PMC2789203

[25] Gallardo C, Soler A, Nieto R, Carrascosa AL, De Mia GM, Bishop RP, Martins C, et al Comparative evaluation of novel African swine fever virus (ASF) antibody detection techniques derived from specific ASF viral genotypes with the OIE internationally prescribed serological tests. Vet Microbiol. 2013; 162: 32–43. 10.1016/j.vetmic.2012.08.011 22944073

[26] Oviedo JM, Rodriguez F, Gomez-Puertas P, Brun A, Gome N, Alonso C, et al High level expression of the major antigenic African swine fever virus proteins p54 and p30 in baculovirus and their potential use as diagnostic reagents. J Virol Methods. 1997; 64: 27–35. 9029527 10.1016/s0166-0934(96)02140-4

[27] Gallardo C, Blanco E, Rodriguez JM, Carrascosa AL, Sánchez-Vizcaíno, JM. Antigenic properties and diagnostic potential of African swine fever virus protein pp62 expressed in insect cells. J Clin Microbiol. 2006; 44: 950–956. 16517882 10.1128/JCM.44.3.950-956.2006 PMC1393094

[28] Pérez-Filgueira DM, Gonzalez-Camacho F, Gallardo C, Resino-Talavan P, Blanco E, Gomez-Casado E, et al Optimization and validation of recombinant serological tests for African swine fever diagnosis based on detection of the p30 protein produced in Trichoplusia ni larvae. J Clin Microbiol. 2006; 44: 3114–3121. 16954235 10.1128/JCM.00406-06 PMC1594705

[29] Gallardo C, Reis AL, Kalema-Zikusoka G, Malta J, Soler A, Blanco E, et al Recombinant antigen targets for serodiagnosis of African swine fever. Clin Vaccine Immunol. 2009; 16: 1012–1020. 10.1128/CVI.00408-08 19420186 PMC2708404

[30] Cubillos C, Gómez-Sebastian S, Moreno N, Nuñez MC, Mulumba-Mfumu LK, Quembo CJ, et al African swine fever virus serodiagnosis: A general review with a focus on the analyses of African serum samples. Virus Res. 2013; 173: 159–167. 10.1016/j.virusres.2012.10.021 23131491

[31] Pang S, Smith J, Onley D, Reeve J, Walker M, Foy C. A comparability study of the emerging protein array platforms with established ELISA procedures. J Immunol Methods. 2005; 302: 1–12. 15993890 10.1016/j.jim.2005.04.007

[32] McVicar JW. Quantitative aspects of the transmission of African swine fever. Am J Vet. Res. 1984; 45: 1535–1541. 6476567

